# Radiogenomic analysis of vascular endothelial growth factor in patients with diffuse gliomas

**DOI:** 10.1186/s40644-019-0256-y

**Published:** 2019-10-21

**Authors:** Zhiyan Sun, Yiming Li, Yinyan Wang, Xing Fan, Kaibin Xu, Kai Wang, Shaowu Li, Zhong Zhang, Tao Jiang, Xing Liu

**Affiliations:** 1 Beijing Neurosurgical Institute, Capital Medical University, 6 Tiantanxili, Beijing, 100050 China; 2 Department of Neurosurgery, Beijing Tiantan Hospital, Capital Medical University, Beijing, China; 3 Chinese Academy of Sciences, Institute of Automation, Beijing, China; 4 Department of Nuclear Medicine, Beijing Tiantan Hospital, Capital Medical University, Beijing, China; 5 Center of Brain Tumor, Beijing Institute for Brain Disorders, Beijing, China; 6 China National Clinical Research Center for Neurological Diseases, Beijing, China; 7 Chinese Glioma Genome Atlas Network (CGGA) and Asian Glioma Genome Atlas Network (AGGA), Beijing, China

**Keywords:** Vascular endothelial growth factor, Diffuse gliomas, Radiomic analysis, Machine learning

## Abstract

**Objective:**

To predict vascular endothelial growth factor (VEGF) expression in patients with diffuse gliomas using radiomic analysis.

**Materials and methods:**

Preoperative magnetic resonance images were retrospectively obtained from 239 patients with diffuse gliomas (World Health Organization grades II–IV). The patients were randomly assigned to a training group (*n* = 160) or a validation group (*n* = 79) at a 2:1 ratio. For each patient, a total of 431 radiomic features were extracted. The minimum redundancy maximum relevance (mRMR) algorithm was used for feature selection. A machine-learning model for predicting VEGF status was then developed using the selected features and a support vector machine classifier. The predictive performance of the model was evaluated in both groups using receiver operating characteristic curve analysis, and correlations between selected features were assessed.

**Results:**

Nine radiomic features were selected to generate a VEGF-associated radiomic signature of diffuse gliomas based on the mRMR algorithm. This radiomic signature consisted of two first-order statistics or related wavelet features (Entropy and Minimum) and seven textural features or related wavelet features (including Cluster Tendency and Long Run Low Gray Level Emphasis). The predictive efficiencies measured by the area under the curve were 74.1% in the training group and 70.2% in the validation group. The overall correlations between the 9 radiomic features were low in both groups.

**Conclusions:**

Radiomic analysis facilitated efficient prediction of VEGF status in diffuse gliomas, suggesting that using tumor-derived radiomic features for predicting genomic information is feasible.

## Introduction

Diffuse gliomas graded from II to IV according to the World Health Organization (WHO) criteria are the most common primary malignant tumors of the brain [1]. Surgical resection combined with radiotherapy and chemotherapy are the main treatments. With the development of precision medicine, the molecular classification of gliomas has become increasingly important with regard to the treatment and prognosis of patients. In 2016, the WHO introduced molecular phenotyping into the classification criteria of tumors of the central nervous system. Accordingly, it is now important to determine the molecular subtype of gliomas prior to treatment [2, 3].

Angiogenesis, which is regulated by vascular endothelial growth factor (VEGF), is a marker of the malignancy of tumor cells. Tumor cells with high expression of VEGF often result in poor prognosis and short survival [4]. In addition, VEGF is a well-known biomarker that is of great significance in the development of tumors, and it is also a promising target in the treatment of gliomas, especially recurrent glioblastomas (GBM) [4–6]. Although anti-angiogenic therapies, such as bevacizumab, have been proved to increase progression-free survival in patients with recurrent GBM, it may not be beneficial for unselected patients [7]. Therefore, the evaluation of VEGF expression held great promising in GBM management.

Magnetic resonance imaging (MRI) is widely used clinically, particularly for the non-invasive imaging of tumors, and it has become one of the most commonly utilized methods for the diagnosis of central nervous system tumors [3, 8]. Notably however, current MRI applications can only analyze the physicochemical characteristics of tumors qualitatively [9, 10]. This ignores a large amount of the digital information in the image. Radiomics is a non-invasive method for extracting textural information from radiological images for analysis and calculation [10–13]. Some studies have applied radiomics technology to tumor analysis and have shown that the approach is feasible [14–16]. Some researchers made a convolutional neural network to determine the Isocitrate dehydrogenase (IDH) mutation status [17], 1p/19q codeletion, and O6-methylguanine-DNA methyltransferase (MGMT) promotor methylation status [18]. Some researchers found the relationship between epidermal growth factor receptor (EGFR) extracellular domain missense mutations and clinical imaging and therapeutic response [19], and established an imaging signature of EGFRvIII [20]. These radiomics approaches have been successfully utilized to predict the genotype of IDH mutation, the expression of EGFR and Ki67 in gliomas [21–23], laying the foundation for detecting VEGF expression status non-invasively.

In the current study, we extracted a large number of radiomics features from preoperative MRI scans of glioma patients with known VEGF expression levels. We hypothesized that a radiomic signature could predict the level of VEGF expression in gliomas via a machine-learning algorithm.

## Materials and methods

### Patients

A total of 239 patients with gliomas were included in this study. All the patients received treatment at Beijing Tiantan Hospital glioma therapy center between June 2010 and September 2012, and met the following criteria: (a) histopathologically confirmed primary glioma, WHO grade II–IV; (b) preoperative T2-weighted magnetic resonance images available; (c) VEGF expression status known; and (d) specific clinical characteristics known (see Additional file 1: Figure S1). A flowchart depicting the exclusion and inclusion of patients is shown in Additional file 1: Figure S1.

The patients were randomly assigned to a training group (*n* = 160) or a validation group (*n* = 79), regardless of VEGF expression level. The random process was performed using the random number generated from the R software. The training group was used to establish a machine-learning model to predict the level of VEGF expression via radiomics features, while the validation group was used to assess the prediction accuracy of the model. Ethics approval for this retrospective study was obtained from the institutional review board of Beijing Tiantan Hospital.

### Clinical characteristics

The median ages of the patients were 43.0 years in the training group and 42.5 years in the validation group. There were 157 males and 82 females in total, and via random assignment 104 men and 56 women were allocated to the training group and 53 men and 26 women were allocated to the validation group. The proportions of patients with low and high VEGF expression were 63/97 in the training group and 27/52 in the validation set. The distributions of characteristics in the two groups were compared using Student’s *t*-test and the Chi-square test, and there were no significant differences in age (*p* = 0.736), sex (*p* = 0.749), tumor grade (*p* = 0.725), VEGF expression level (*p* = 0.435), or tumor location (*p* = 0.860). Detailed information pertaining to the clinical characteristics of the patients is shown in Table 1.

**Table 1:** Patient characteristics

	Training (*n* = 160)	Validation (*n* = 79)	*p* value
Age (years; mean)	43.0	42.5	0.736^a^
Sex (male/female)	104/56	53/26	0.749^b^
Grade II/Grade III/Grade IV	75/43/42	39/23/17	0.725^b^
Low VEGF/High VEGF	63/97	27/52	0.435^b^
Tumor location left/right	87/73	42/37	0.860^b^

^a^Student’s *t*-test, ^b^Chi-square test

### Data acquisition and region of interest segmentation

MRI scans were performed using 3.0-T scanners (179 patients: Trio, 3.0-T, Siemens; 60 patients: sigma, 3.0-T, GE). Tumor regions of interest (ROIs) were only segmented on T2-weighted (T2) images because II-IV grades of gliomas were included in the current study (identifying tumor borders of low-grade gliomas is hard on T1-weighted and contrast-enhanced sequences). The parameters used to acquire T2 images were repetition time 4500~6000 ms, echo time 84~122.5 ms, section thickness 3~5 mm, field of view of (180~240) mm × (219~256) mm, and matrix size (160~512) × (208~512) pixels. ROIs were manually delineated by two neuroradiologists (with eight- and 10-year work experience in the field of neuroradiology, respectively) on T2 images using the MRIcron software (http://www.mccauslandcenter.sc.edu/mricro). ROIs on the T2 image were defined as edema area according to previous literature [24, 25]. A third senior neuroradiologist with 10 years work experience then reevaluated the ROIs and made final decisions in cases where there was a lack of consensus.

### Feature extraction

To reduce bias due to data heterogeneity, the intensities of the voxels in each image were normalized to the z distribution. The slice thickness of MRI was resampled to 1 mm before feature extraction. Extraction of quantitative radiomic features was conducted as previously described [26], and the detailed equation pertaining to each feature is presented as Additional file 3 in that previous report. The feature extraction happened in 3D, and as a forward pass. We only extract radiomic features from preoperative T2 sequences, and a total of 431 radiomic features were obtained. The feature set included 14 first order statistics (pertaining to the distribution of signal intensity of images), 8 shape and size-based features (quantifying the shape and size of tumors), 33 textural features (pertaining to intratumoral heterogeneity), and 376 wavelet features that were derived from group 1 and group 3 features via wavelet decomposition (using directional low-pass and high-pass filtering, the original feature was decomposed into 8 decompositions). All feature extraction processes were conducted using software developed in-house and implemented in MATLAB (2014a). The detailed function of the radiomic features are listed in the Additional file 3, and the extracted features are in compliance with the Image Biomarker Standardization Initiative [27, 28].

### Immunohistochemistry

VEGF expression levels were evaluated by an eight-year work experience pathologist using typical tumor samples collected from the patients. Immunostaining was performed using an anti-VEGF antibody (Santa Cruz Biotechnology, Santa Cruz, CA) at a dilution of 1:100 in accordance with the manufacturer’s instructions. Briefly, formalin-fixed paraffin-embedded tissue sections were cut into 5-μm sections, which were then dried, dewaxed in xylene, rinsed in graded ethanol, and rehydrated in double-distilled water. Two pathologists who were blind to the clinical data scored the degree of staining. VEGF expression level was scored according to clinical practice: (−) represented no or rare expression (< 5% positive cells); (+) represented mild expression (6–25% positive cells); (++) represented moderate expression (26–50% positive cells); and (+++) represented strong expression (> 50% positive cells). Low VEGF expression was defined as VEGF (− and +), and high VEGF expression was defined as VEGF (++ and +++) [29].

### Feature selection and classification

**Fig. 1:**
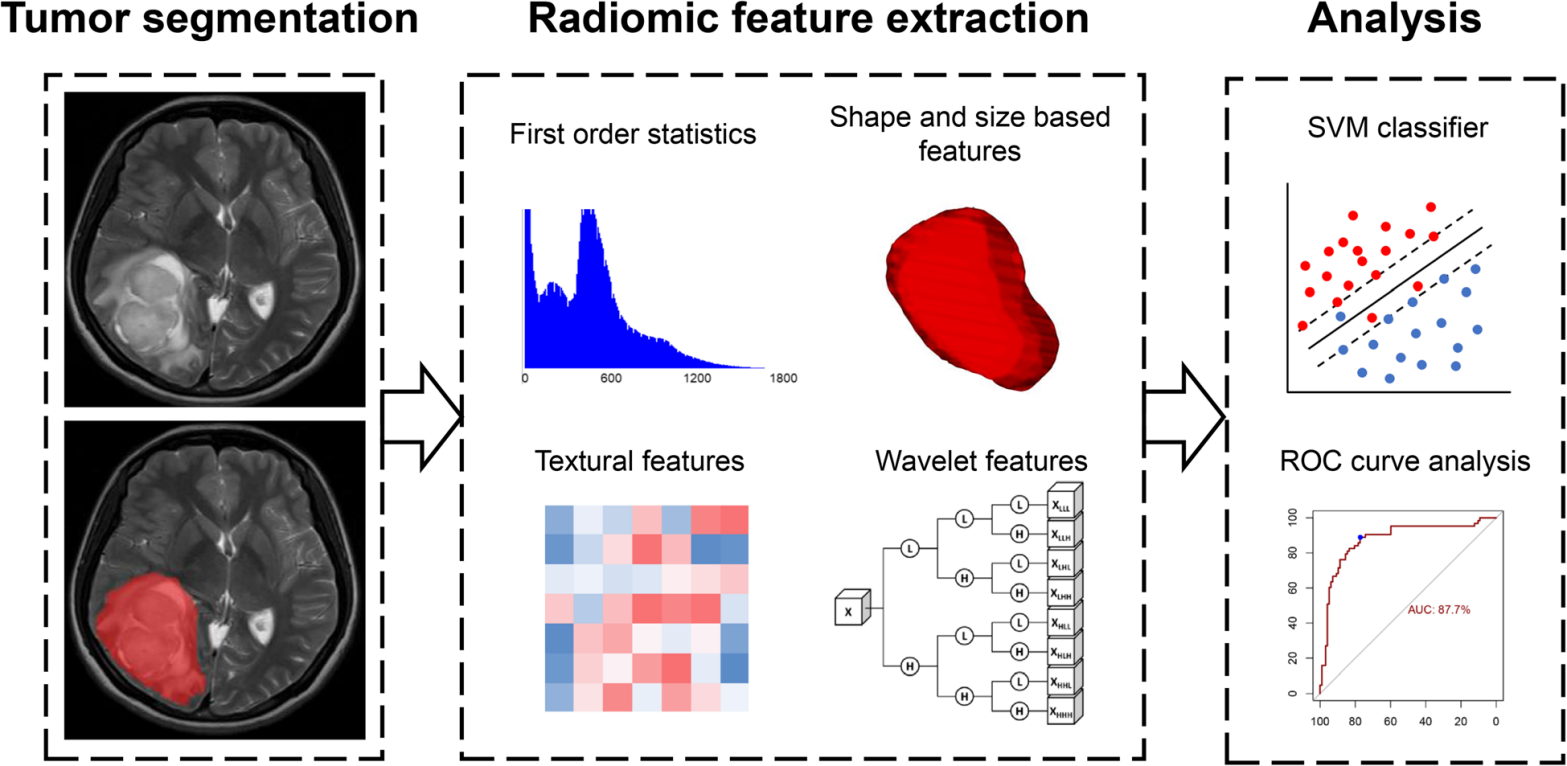
The radiomics protocol. After the acquisition of magnetic resonance images, tumor segmentation was conducted using the image data. High-throughput radiomic features were then extracted from the segmented tumors, and further radiomic analysis was performed using these extracted features. SVM, support vector machine; ROC, receiver operating characteristic

To establish the radiomics model, the minimum redundancy maximum relevance (mRMR) algorithm was applied to select a subset of features from the 431 extracted radiomic features. The mRMR algorithm is an efficient data screening tool that has been widely used in many previous studies [30, 31]. In addition, we utilized a support vector machine (SVM) classifier to establish a machine-learning model for VEGF prediction. The SVM classifier is a widely used pattern recognition tool [32, 33]. Based on 10-fold cross validation, the parameters of SVM classifier were determined with the grid-search: kernel = radial, gamma = {10^− 4^, 10^− 3^, 10^− 2^, 10^− 1^, 1, 10, 10^2^, 10^3^, 10^4^}, cost = {10^− 4^, 10^− 3^, 10^− 2^, 10^− 1^, 1, 10, 10^2^, 10^3^, 10^4^}. Via these methods, we established a radiomics-based signature prediction model using data from the training group, and applied the same model in the validation group. A radiomics analysis protocol is shown in Fig. 1.

### Statistical analysis

Statistical analyses and figure generation were mainly performed using R software (version 3.3.2; https://www.r-project.org/). The mRMR and SVM algorithms were conducted using “mRMRe” and “e1071” packages, respectively. Receiver operating characteristic (ROC) curves, and correlation heatmaps were depicted using “pROC” and “corrplot” packages, respectively. Differences between clinical characteristics were evaluated using the Chi-square test or Student’s *t*-test.

## Results

### Feature selection and classification

In the current study, an efficient feature selection tool known as the mRMR algorithm was used, and a subset of 9 features were screened from a total of 431 radiomic features. The names and descriptions of these 9 selected features are shown in Table 2.

**Table 2:** Nine radiomic features selected by the minimum redundancy maximum relevance algorithm

Number	Features	Description
1	Cluster Tendency_HLL	One of the wavelet features derived from Cluster Tendency. Cluster Tendency is a measure of groupings of voxels with similar gray-level values.
2	Entropy_LLL (group 1 derived)	One of the wavelet features derived from Entropy. Entropy specifies the uncertainty/randomness in the image values.
3	Long Run Low Gray Level Emphasis_LHL	One of the wavelet features derived from Long Run Low Gray Level Emphasis. Long Run Low Gray Level Emphasis measures the joint distribution of long runs and low gray level values.
4	Minimum	Minimum describes the minimum signal intensity.
5	Short Run High Gray Level Emphasis_LLH	One of the wavelet features derived from Short Run High Gray Level Emphasis. Short Run High Gray Level Emphasis measures the joint distribution of short runs and high gray level values.
6	Short Run Low Gray Level Emphasis_LLL	One of the wavelet features derived from Short Run Low Gray Level Emphasis. Short Run Low Gray Level Emphasis measures the joint distribution of short runs and low gray level values.
7	Short Run Low Gray Level Emphasis_LHH	One of the wavelet features derived from Short Run Low Gray Level Emphasis. Short Run Low Gray Level Emphasis measures the joint distribution of short runs and low gray level values.
8	Short Run Low Gray Level Emphasis_HLL	One of the wavelet features derived from Short Run Low Gray Level Emphasis. Short Run Low Gray Level Emphasis measures the joint distribution of short runs and low gray level values.
9	Short Run Low Gray Level Emphasis_HLH	One of the wavelet features derived from Short Run Low Gray Level Emphasis. Short Run Low Gray Level Emphasis measures the joint distribution of short runs and low gray level values.

**Fig. 2:**
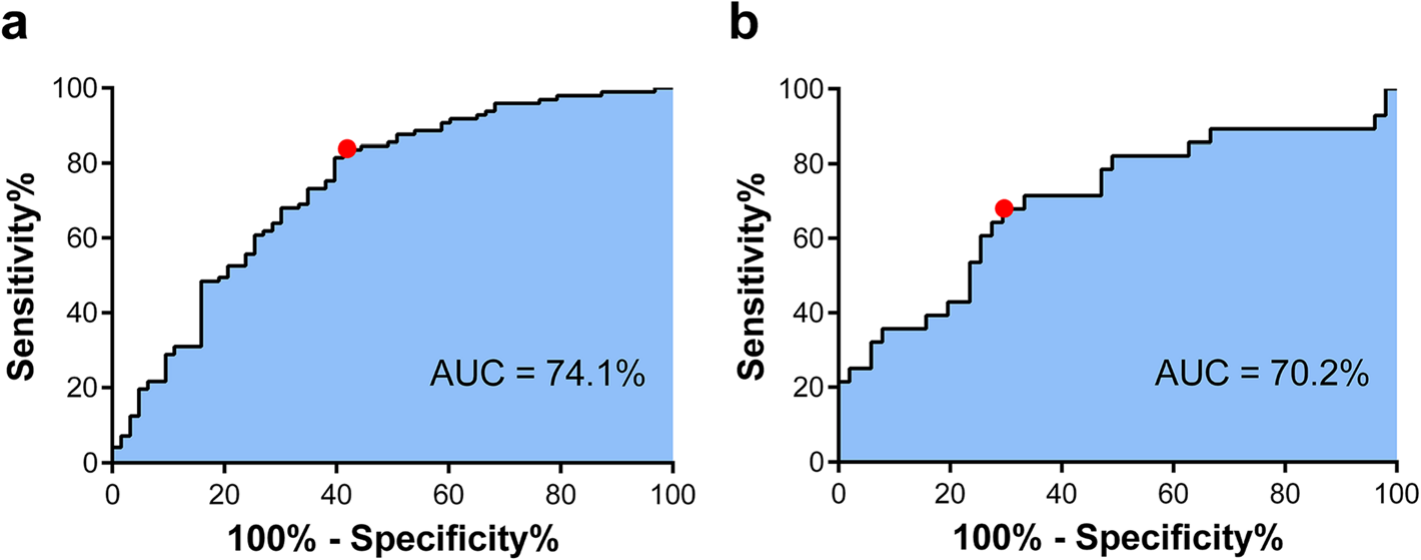
Receiver operating characteristic curves for vascular endothelial growth factor status prediction in the training group and the validation group. **a** In the training group, the area under the curve was 74.1%. At the optimal cutoff value (−0.356), the respective sensitivity, specificity, and accuracy values were 83.5, 58.7, and 71.3% (red dot). **b** In the validation set, the area under the curve was 70.2% and the optimal cutoff value (− 0.570) exhibited respective sensitivity, specificity, and accuracy values of 67.9, 70.6, and 72.3% (red dot). AUC, area under the curve

According to the cross-validation process, the SVM classifier performed best when the parameter gamma = 10^− 3^ and cost = 10^− 3^. Based on the selected radiomic features and the SVM classifier, a VEGF predictive machine-learning model was built using data derived from the training group. The areas under curve (AUC) were 0.741 in the training group (Fig. 2a) and 0.702 in the validation group (Fig. 2b). In ROC curve analysis, in the training group the optimal cutoff point of − 0.356 exhibited respective sensitivity, specificity, and accuracy values of 83.5, 58.7, and 71.3%, and in validation group the optimal cutoff point of − 0.570 yielded corresponding values of 67.9, 70.6, and 72.3%. Hence, the 9 selected radiomics features could be regarded as a VEGF-related signature, and the model established via mRMR and SVM algorithms in the training group exhibited effective performance in the validation group.

### Correlations between selected features

**Fig. 3:**
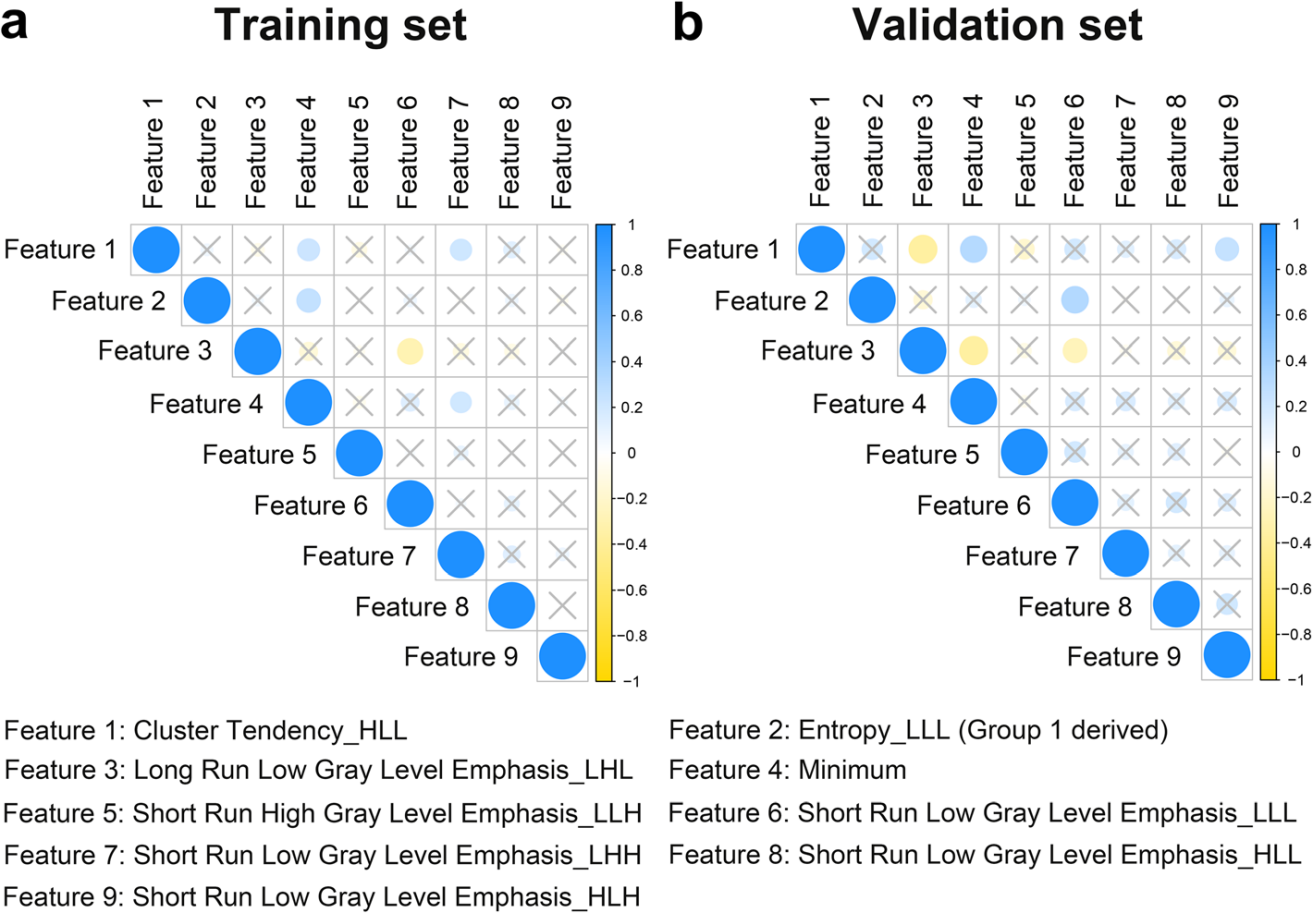
Correlations between the 9 radiomic features that constituted the vascular endothelial growth factor-associated radiomic signature in the training and validation groups. Yellow dots indicate positive correlations and blue dots indicate negative correlations. Different sizes and color depths of dots indicate different correlation coefficients. Dots covered with crosses indicate non-significant correlations (*p* > 0.05)

Correlations between the VEGF-related features in the training group and the validation group are shown in Fig. 3. Although some of the 9 features exhibited high positive and negative correlations, the overall correlations between the features were low (mean ± standard deviation = 0.112 ± 0.029 in the training group and 0.174 ± 0.052 in the validation group), suggesting that the features were independent of each other.

## Discussion

In the current study, VEGF-related radiological signatures were analyzed via feature data extracted from T2-weighted images of patients with diagnosed gliomas. Image features were extracted from each sequence and used to establish a radiomics-based model to predict VEGF expression levels via an mRMR algorithm and SVM classifier. The model achieved AUCs of 74.1% in the training group and 70.2% in the validation group. Overall, the results suggest that VEGF expression can be predicted using non-invasive radiological data, and that a machine-learning approach that integrates multivariate features is more effective than using individual features.

It has been reported that VEGF status was associated with some imaging features [29, 34–38]. A previous study reported that gliomas of different VEGF status tended to be situated at different locations [39]. They revealed that glioblastomas with high VEGF expression were more likely located in the left frontal lobe and the right caudate and these with low VEGF expression were more frequently located in the posterior region of the right lateral ventricle. We validated their findings on 59 patients in our study cohort, since only the 59 patients were diagnosed as glioblastoma. As shown in Additional file 2: Figure S2, the overall predictive accuracy was 60%. Notably, there were 11 patients with tumors that did not involve left frontal lobe or the right caudate or the posterior region of the right lateral ventricle, which could not be classified. This kind of condition would limit the application of this method.

Moreover, Awasthi R et al. found that relative cerebral blood volume and relative cerebral blood flow were significantly correlated with VEGF expression level [38]; Li K et al. reported that combining the radiomic features and VEGF expression level could predict pelvic lymphatic metastasis [34]; Yin Q et al. revealed the associations between tumor angiogenesis and radiomic imaging features from PET/MRI [35], and Beig N et al. analyzed the hypoxia pathway radiomic features and predicted the overall survival in GBM [36]. These studies show that there were some radiological features that were significantly associated with VEGF expression status, but they did not use independent validation data-sets to evaluate their findings, and their predictive power is limited. In recent years, radiomic analysis combined with algorithms has been widely used in radiology studies, and constitutes an efficient tool for studying relationships between images and tumors [11]. Many researchers have proved that radiomics models can predict molecule expression status efficiently [40–42]. Radiomics was also used to predict the anti-angiogenic treatment response [37]. We believe that there are clinically useful relationships between radiomic features and VEGF expression status.

The model established in the present study performed effectively in both the training group and the validation group, affirming the hypothesis that radiomics can predict VEGF expression level in tumor tissues. A previous study indicated that entropy could serve as an indicator for intra-tumoral heterogeneity and the degree of tumor malignancy [43], which could be used as an example to explain the association between radiomic features and VEGF expression. In addition, previous studies have shown that glioma patients with high expression of VEGF are more likely to have tissue edema [44–47]. T2 sequences can reflect tumors and tissue edema more accurately [48, 49]. This may be the reason why the features selected based on T2 sequences could effectively establish a VEGF prediction model.

To establish the radiomics model, the mRMR algorithm was applied to select a subset of 9 features from the 431 extracted radiomic features. The mRMR algorithm is an efficient data dimensionality reduction algorithm for finding a set of both relevant and independent features that is widely used in bioinformation analysis [30, 31, 50]. The curse of dimensionality could be solved using the mRMR algorithm. The SVM classifier is an effective tool that exhibits better performance than other algorithms with regard to pattern recognition [32, 51, 52]. In the present study, the mRMR algorithm was used in combination with the SVM classifier to develop a method capable of effectively predicting VEGF expression status in glioblastoma patients.

The current study had some limitations. All the images were generated at a single center, so a multi-center study needs to be conducted in the future to investigate the universality of the model. Additionally, the correlative nature of the reported radiomic signature to VGEF expression needs to be further investigated by comparing to tissue biopsy results*.* It was also a retrospective study, so a prospective study is needed to verify the accuracy of the prediction model. Cases are manually annotated. In our current work we are trying to use an automated segmented approach that can level up the speed of radiomic pipeline. Next, we would collect more MRI protocols such as T1W and FLAIR (additionally also MRS, DTI) in our further work to build an advanced model. Finally, MRI scans have not been post-processed to a standard atlas, which might make the reproducibility of results difficult.

## Conclusion

In conclusion, in the present study there were significant correlations between VEGF expression level and radiomic features in gliomas. Using the mRMR algorithm and SVM classifier, a VEGF expression level radiomic signature was developed, and VEGF expression level was effectively predicted in both a training group and a validation group. Radiogenomic analysis showed that VEGF expression level could also be reflected by the radiomic signature extracted from radiological images, indicating that the radiomic approach could potentially be a noninvasive surrogate indicator of gene expression level, and further assist patient-tailored treatment.

## Supplementary information

**Additional file 1: Figure S1.** Flow diagram of patients included and excluded in the final analysis.

**Additional file 2: Figure S2.** The prediction results of VEGF expression status in our cohort. The blue bars indicate that the predicted VEGF expression status was in accordance with the true VEGF expression status. The yellow bars indicate that the predicted VEGF expression status is not consistent with the true VEGF expression status.

**Additional file 3.** The feature extraction method used in the current study.

## Data Availability

The datasets supporting the conclusion of this article available from the corresponding author on reasonable request.

## Abbreviations

AUC: Areas under curve
MRI: Magnetic resonance imaging
mRMR: the minimum redundancy maximum relevance algorithm
ROC: Receiver operating characteristic curves
ROI: Regions of interest
SVM: Support vector machine
T2: T2-weighted
VEGF: Vascular endothelial growth factor
