# Supplementary figures and images for: Radiogenomic analysis of vascular endothelial growth factor in patients with diffuse gliomas
Source: Cancer Imaging. 2019 Oct 21;19:68. doi: 10.1186/s40644-019-0256-y (PMC6805458; doi:10.1186/s40644-019-0256-y)

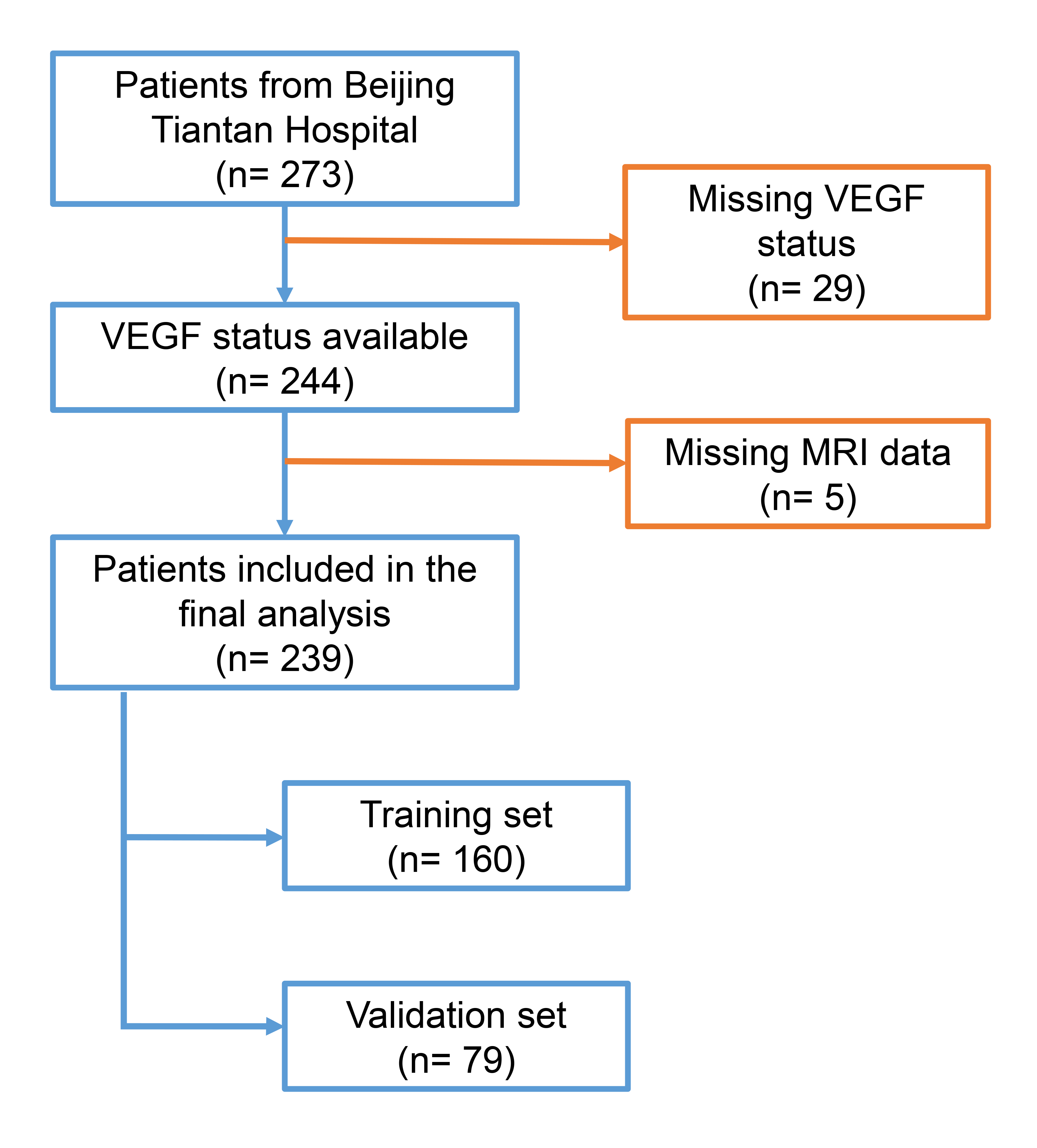

Supplement: Supplementary file 1 — Additional file 1: Figure S1. Flow diagram of patients included and excluded in the final analysis. [file 40644_2019_256_MOESM1_ESM.tif]

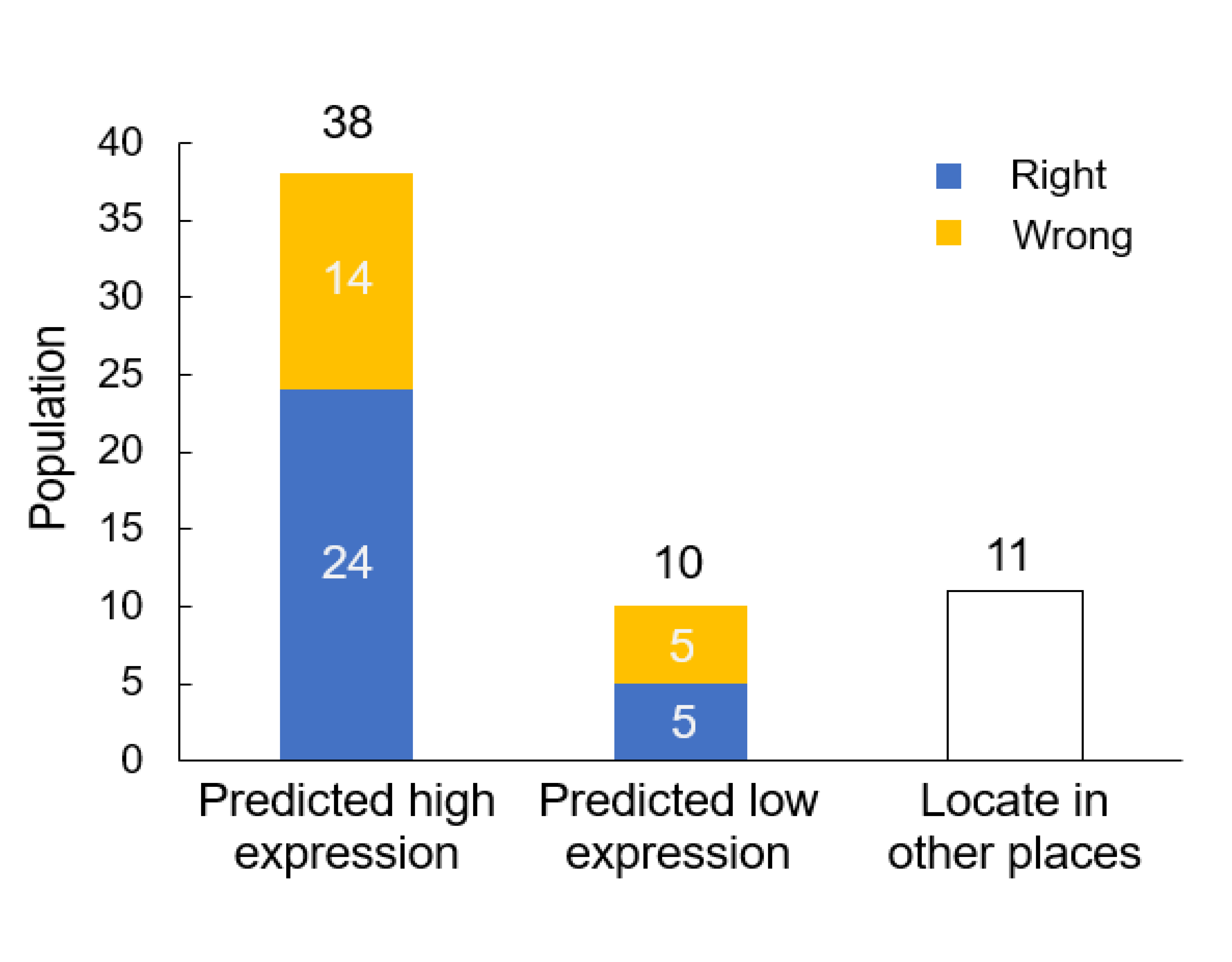

Supplement: Supplementary file 2 — Additional file 2: Figure S2. The prediction results of VEGF expression status in our cohort. The blue bars indicate that the predicted VEGF expression status was in accordance with the true VEGF expression status. The yellow bars indicate that the predicted VEGF expression status is not consistent with the true VEGF expression status. [file 40644_2019_256_MOESM2_ESM.tif]
