# Supplementary material for: Radiogenomic analysis of vascular endothelial growth factor in patients with diffuse gliomas
Source: Cancer Imaging. 2019 Oct 21;19:68. doi: 10.1186/s40644-019-0256-y (PMC6805458; doi:10.1186/s40644-019-0256-y)
Supplement: Supplementary file 3 — Additional file 3. The feature extraction method used in the current study. [file 40644_2019_256_MOESM3_ESM.docx]

Additional file

Totally, we extracted 431 features according to the previous study by Aerts HJWL et al [[1](#_ENREF_1)]. These features could be divided into four groups as follows:

1. First order statistics (14 features)
2. Shape and size-based features (8 features)
3. Textural features (33 features)
4. Wavelet features (376 features)

***Group 1. First order statistics***

First-order statistics describe the voxel intensities distribution within the image through commonly used and basic metrics. $X$ represents the three-dimensional image matrix with $N$ voxels and $P$ represents the first order histogram divided by $N_{l}$ discrete intensity levels. The following first order statistics were extracted:

1. **Energy:**

$$energy=\sum_{i}^{N} {X(i)}^{2}$$

2. **Entropy:** $entropy=-\sum_{i=1}^{N_{l}} P(i)\log_{2} P(i)$
3. **Kurtosis:** $kurtosis=\frac{\frac{1}{N}\sum_{i=1}^{N} {(X\left( i \right)-\bar{X})}^{4}}{\left( \sqrt{\frac{1}{N}\sum_{i=1}^{N} \left( X\left( i \right)-\bar{X} \right)^{2}} \right)^{2}}$ $\bar{X}$ represents the mean of $X$.
4. **Maximum:**The maximum intensity value of $X$.
5. **Mean:** $mean=\frac{1}{N}\sum_{i}^{N} X(i)$
6. **Mean absolute deviation:**

The mean of the absolute deviations of all voxel intensities around the mean intensity value.

1. **Median:**The median intensity value of $X$.
2. **Minimum:**The minimum intensity value of $X$.
3. **Range:**The range of intensity values of $X$.
4. **Root mean square (RMS):**

$$RMS=\sqrt{\frac{\sum_{i}^{N} {X(i)}^{2}}{N}}$$

5. **Skewness:** $skewness=\frac{\frac{1}{N}\sum_{i=1}^{N} {(X\left( i \right)-\bar{X})}^{3}}{\left( \sqrt{\frac{1}{N}\sum_{i=1}^{N} {(X\left( i \right)-\bar{X})}^{2}} \right)^{3}}$ $\bar{X}$ represents the mean of $X$.
6. **Standard deviation:**

$$standard deviation=\left( \frac{1}{N-1}\sum_{i=1}^{N} {(X\left( i \right)-\bar{X})}^{2} \right)^{1/2}$$

$\bar{X}$ represents the mean of $X$.

1. **Uniformity:**

$$uniformity=\sum_{i=1}^{N_{l}} {P(i)}^{2}$$

2. **Variance:** $variance=\frac{1}{N-1}\sum_{i=1}^{N} {(X\left( i \right)-\bar{X})}^{2}$ $\bar{X}$ represents the mean of $X$.

The standard deviation, variance and mean absolute deviation are measures of the histogram dispersion, that is, a measure of how much the gray levels differ from the mean. The variance, skewness and kurtosis are the most frequently used central moments. The skewness measures the degree of histogram asymmetry around the mean, and kurtosis is a measure of the histogram sharpness. As measures of histogram randomness, we computed the uniformity and entropy of the image histogram.

***Group 2. Shape and size-based features***

This feature group contains descriptors of the three-dimensional size and shape of the tumor region. These features describe how spherical, rounded, or elongated the shape of the tumor is. $V$ represents the volume and $A$ the surface area of the volume of interest. Here are the shape and size-based features:

1. **Compactness 1**:

$$compactness 1=\frac{V}{\sqrt{\pi}A^{\frac{2}{3}}}$$

2. **Compactness 2**:

$$compactness 2=36\pi\frac{V^{2}}{A^{3}}$$

3. **Maximum 3D diameter**:

The maximum three-dimensional tumor diameter calculates the largest pairwise Euclidean distance, between voxels on the surface of the tumor volume.

1. **Spherical disproportion**:

$$spherical disproportion=\frac{A}{4\pi R^{2}}$$

$R$ is the radius of a sphere with the same volume as the tumor.

1. **Sphericity**:
$sphericity=\frac{\pi^{\frac{1}{3}}{(6V)}^{\frac{2}{3}}}{A}$
2. **Surface area:**

The surface area is measured by triangulation (i.e. dividing the surface into connected triangles) and is defined as:

$$A=\sum_{i=1}^{N} \frac{1}{2}\left| a_{i}b_{i}\times a_{i}c_{i} \right|$$

 
$N$ is the total number of triangles covering the surface and $a$, $b$ and $c$ are edge vectors of the triangles.

1. **Surface to volume ratio**:
$surface to volume ratio=\frac{A}{V}$
2. **Volume:**The tumor volume ($V)$ is determined by counting the number of pixels in the tumor region and multiplying this value by the voxel size.

***Group 3. Textural features***

The features shown above that resulted from group 1 (first-order statistics) provide information related to the gray-level distribution of the image; however, they do not provide any information regarding the relative position of the various gray levels over the image. In this group we therefore included textural features describing patterns or the spatial distribution of voxel intensities, which were calculated from respectively gray level co-occurrence (GLCM)^1^[[2](#_ENREF_2)][[2](#_ENREF_2)]^221^, gray level run-length (GLRLM)^2^ and gray level size (GLSZM)^3^ texture matrices. Determining texture matrix representations requires the voxel intensity values within Value of Interest (VOI) to be discretized. Voxel intensities were therefore resampled into equally spaced bins using a bin-width of 25 Hounsfield Units. This discretization step not only reduces image noise, but also normalizes intensities across all patients, allowing for a direct comparison of all calculated textural features between patients. Texture matrices were determined considering 26-connected voxels (i.e. voxels were considered to be neighbors in all 13 directions in three dimensions).

*Gray-Level Co-Occurrence Matrix based features*

A GLCM is defined as $P(i,j;\delta,\alpha)$, a matrix with size $N_{g}\times N_{g}$ describing the second-order joint probability function of an image, where the $(i,j)$th element represents the number of times the combination of intensity levels $i$ and $j$ occur in two pixels in the image, that are separated by a distance of $\delta$ pixels in direction $\alpha$, and $N_{g}$ is the number of discrete gray level intensities. As a two dimensional example, let the following matrix represent a 5x5 image, having 5 discrete gray levels:

$$I=\begin{matrix} 1 & 2 & 5 & 2 & 3 \\ 3 & 2 & 1 & 3 & 1 \\ 1 & 3 & 5 & 5 & 2 \\ 1 & 1 & 1 & 1 & 2 \\ 1 & 2 & 4 & 3 & 5 \end{matrix}$$

For distance $\delta=1$ (considering pixels with a distance of 1 pixel from each other) in direction $\alpha=0$, where 0 degrees is the horizontal direction, the following GLCM is obtained:

$$P(1,0)=\begin{matrix} 3 & 3 & 2 & 0 & 0 \\ 1 & 0 & 1 & 1 & 1 \\ 0 & 1 & 0 & 0 & 2 \\ 0 & 0 & 1 & 0 & 0 \\ 0 & 2 & 0 & 0 & 1 \end{matrix}$$

In this study, distance $\delta$ was set to 1 and direction $\alpha$ to each of the 13 directions in three dimensions, yielding a total of 13 gray level co-occurrence matrices for each 3D image. From these gray-level co-occurrence matrices, several textural features are derived. Each 3D gray level co-occurrence-based feature was then calculated as the mean of the feature calculations for each of the 13 directions.

Let:

$P(i,j)$ be the co-occurrence matrix for an arbitrary $\delta$ and $\alpha$,

$N_{g}$ be the number of discrete intensity levels in the image,

$\mu$ be the mean of $P(i,j)$,

$p_{x}\left( i \right)=\sum_{j=1}^{N_{g}} P(i,j)$ be the marginal row probabilities,

$p_{y}\left( i \right)=\sum_{i=1}^{N_{g}} P(i,j)$ be the marginal column probabilities,

$\mu_{x}$ be the mean of $p_{x}$,

$\mu_{y}$ be the mean of $p_{y}$,

$\sigma_{x}$ be the standard deviation of $p_{x}$,

$\sigma_{y}$ be the standard deviation of $p_{y}$,

$p_{x+y}\left( k \right)=\sum_{i=1}^{N_{g}} \sum_{j=1}^{N_{g}} P\left( i,j \right)$, $i+j=k$, $k=2,3,\ldots,2N_{g}$,

$p_{x-y}\left( k \right)=\sum_{i=1}^{N_{g}} \sum_{j=1}^{N_{g}} P\left( i,j \right)$, $\left| i-j \right|=k$, $k=0,1,\ldots,N_{g}-1$,

$HX=-\sum_{i=1}^{N_{g}} p_{x}(i)\log_{2} \left[ p_{x}(i) \right]$ be the entropy of $p_{x}$,

$HY=-\sum_{i=1}^{N_{g}} p_{y}(i)\log_{2} \left[ p_{y}(i) \right]$ be the entropy of $p_{y}$,

$H=-\sum_{i=1}^{N_{g}} \sum_{j=1}^{N_{g}} P\left( i,j \right)\log_{2} \left[ P\left( i,j \right) \right]$ be the entropy of $P(i,j)$,

$HXY1=-\sum_{i=1}^{N_{g}} \sum_{j=1}^{N_{g}} P\left( i,j \right)log(p_{x}\left( i \right)p_{y}\left( j \right))$,

$HXY2=-\sum_{i=1}^{N_{g}} \sum_{j=1}^{N_{g}} p_{x}\left( i \right)p_{y}(j)log(p_{x}\left( i \right)p_{y}\left( j \right))$.

1. **Autocorrelation:**

$$autocorrelation=\sum_{i=1}^{N_{g}} \sum_{j=1}^{N_{g}} ijP(i,j)$$

2. **Cluster Prominence:**

$$cluster prominence=\sum_{i=1}^{N_{g}} \sum_{j=1}^{N_{g}} \left[ i+j-\mu_{x}(i)-\mu_{y}(j) \right]^{4}P(i,j)$$

3. **Cluster Shade:**

$$cluster shade=\sum_{i=1}^{N_{g}} \sum_{j=1}^{N_{g}} \left[ i+j-\mu_{x}(i)-\mu_{y}(j) \right]^{3}P(i,j)$$

4. **Cluster Tendency:**

$$cluster tendency=\sum_{i=1}^{N_{g}} \sum_{j=1}^{N_{g}} \left[ i+j-\mu_{x}\left( i \right)-\mu_{y}\left( j \right) \right]^{2}P(i,j)$$

5. **Contrast:**

$$contrast=\sum_{i=1}^{N_{g}} \sum_{j=1}^{N_{g}} \left| i-j \right|^{2}P(i,j)$$

6. **Correlation:**

$$correlation=\frac{\sum_{i=1}^{N_{g}} \sum_{j=1}^{N_{g}} ijP\left( i,j \right)-\mu_{i}{\left( i \right)\mu}_{j}(j)}{\sigma_{x}(i)\sigma_{y}(j)}$$

7. **Difference entropy:**

$$difference entropy=\sum_{i=0}^{N_{g}-1} P_{x-y}(i)\log_{2} \left[ P_{x-y}(i) \right]$$

8. **Dissimilarity:**

$$dissimilarity=\sum_{i=1}^{N_{g}} \sum_{j=1}^{N_{g}} \left| i-j \right|P(i,j)$$

9. **Energy:**

$$energy=\sum_{i=1}^{N_{g}} \sum_{j=1}^{N_{g}} \left[ P(i,j) \right]^{2}$$

10. **Entropy ($\boldsymbol{H}$):**

$$entropy=-\sum_{i=1}^{N_{g}} \sum_{j=1}^{N_{g}} P\left( i,j \right)\log_{2} \left[ P\left( i,j \right) \right]$$

11. **Homogeneity 1:**

$$homogeneity 1=\sum_{i=1}^{N_{g}} \sum_{j=1}^{N_{g}} \frac{P(i,j)}{1+\left| i-j \right|}$$

12. **Homogeneity 2:**

$$homogeneity 2=\sum_{i=1}^{N_{g}} \sum_{j=1}^{N_{g}} \frac{P(i,j)}{1+\left| i-j \right|^{2}}$$

13. **Informational measure of correlation 1 (IMC1):**

$$IMC1=\frac{HXY-HXY1}{\text{max}\left\{ HX,HY \right\}}$$

14. **Informational measure of correlation 2 (IMC2):**

$$IMC2=\sqrt{1-e^{-2(HXY2-HXY)}}$$

15. **Inverse Difference Moment Normalized (IDMN):**

$$IDMN=\sum_{i=1}^{N_{g}} \sum_{j=1}^{N_{g}} \frac{P(i,j)}{1+\left( \frac{\left| i-j \right|^{2}}{N^{2}} \right)}$$

16. **Inverse Difference Normalized (IDN):**

$$IDN=\sum_{i=1}^{N_{g}} \sum_{j=1}^{N_{g}} \frac{P(i,j)}{1+\left( \frac{\left| i-j \right|}{N} \right)}$$

17. **Inverse variance:**

$$\begin{matrix} inverse variance=\sum_{i=1}^{N_{g}} \sum_{j=1}^{N_{g}} \frac{P(i,j)}{\left| i-j \right|^{2}} & ,i\neq j \end{matrix}$$

18. **Maximum Probability:**

$$maximum probability=\text{max}\left\{ P(i,j) \right\}$$

19. **Sum average:**

$$sum average=\sum_{i=2}^{2N_{g}} \left[ iP_{x+y}(i) \right]$$

20. **Sum entropy:**

$$sum entropy=-\sum_{i=2}^{2N_{g}} P_{x+y}(i)\log_{2} \left[ P_{x+y}(i) \right]$$

21. **Sum variance:**

$$sum variance=\sum_{i=2}^{2N_{g}} {(i-SE)}^{2}P_{x+y}(i)$$

22. **Variance:**

$$variance=\sum_{i=1}^{N_{g}} \sum_{j=1}^{N_{g}} {(i-\mu)}^{2}P(i,j)$$

*Gray-Level Run-Length matrix based features*

Run length metrics quantify gray level runs in an image. A gray level run is defined as the length in number of pixels, of consecutive pixels that have the same gray level value. In a gray level run length matrix $p(i,j|\theta)$, the $(i,j)$th element describes the number of times $j$ a gray level $i$ appears consecutively in the direction specified by $\theta$, and $N_{g}$ is the number of discrete gray level intensities. As a two-dimensional example, consider the following 5x5 image, with 5 discrete gray levels:

$$I=\begin{matrix} 5 & 2 & 5 & 4 & 4 \\ 3 & 3 & 3 & 1 & 3 \\ 2 & 1 & 1 & 1 & 3 \\ 4 & 2 & 2 & 2 & 3 \\ 3 & 5 & 3 & 3 & 2 \end{matrix}$$

The GLRL matrix for $\theta=0$, where 0 degrees is the horizontal direction, then becomes:

$$p\left( 0 \right)=\begin{matrix} 1 & 0 & 1 & 0 & 0 \\ 3 & 0 & 1 & 0 & 0 \\ 4 & 1 & 1 & 0 & 0 \\ 1 & 1 & 0 & 0 & 0 \\ 3 & 0 & 0 & 0 & 0 \end{matrix}$$

In this study, a GLRL matrix was computed for every of the 13 directions in three dimensions, from which the below textural features were derived. Each 3D GLRL feature was then calculated as the mean of the feature values for each of the 13 directions.

Let:

$p(i,j|\theta)$ be the $\left( i,j \right)$th entry in the given run-length matrix $p$ for a direction $\theta$,

$N_{g}$ the number of discrete intensity values in the image,

$N_{r}$ the number of different run lengths,

$N_{p}$ the number of voxels in the image.

1. **Short Run Emphasis (SRE)**

$$SRE=\frac{\sum_{i=1}^{N_{g}} \sum_{j=1}^{N_{r}} \left[ \frac{p(i,j|\theta)}{j^{2}} \right]}{\sum_{i=1}^{N_{g}} \sum_{j=1}^{N_{r}} p(i,j|\theta)}$$

2. **Long Run Emphasis (LRE)**

$$LRE=\frac{\sum_{i=1}^{N_{g}} \sum_{j=1}^{N_{r}} j^{2}p(i,j|\theta)}{\sum_{i=1}^{N_{g}} \sum_{j=1}^{N_{r}} p(i,j|\theta)}$$

3. **Gray Level Non-Uniformity (GLN)**

$$GLN=\frac{\sum_{i=1}^{N_{g}} \left[ \sum_{j=1}^{N_{r}} p(i,j|\theta) \right]^{2}}{\sum_{i=1}^{N_{g}} \sum_{j=1}^{N_{r}} p(i,j|\theta)}$$

4. **Run Length Non-Uniformity (RLN)**

$$RLN=\frac{\sum_{j=1}^{N_{r}} \left[ \sum_{i=1}^{N_{g}} p(i,j|\theta) \right]^{2}}{\sum_{i=1}^{N_{g}} \sum_{j=1}^{N_{r}} p(i,j|\theta)}$$

5. **Run Percentage (RP)**

$$RP=\sum_{i=1}^{N_{g}} \sum_{j=1}^{N_{r}} \frac{p(i,j|\theta)}{N_{p}}$$

6. **Low Gray Level Run Emphasis (LGLRE)**

$$LGLRE=\frac{\sum_{i=1}^{N_{g}} \sum_{j=1}^{N_{r}} \left[ \frac{p(i,j|\theta)}{i^{2}} \right]}{\sum_{i=1}^{N_{g}} \sum_{j=1}^{N_{r}} p(i,j|\theta)}$$

7. **High Gray Level Run Emphasis (HGLRE)**

$$HGLRE=\frac{\sum_{i=1}^{N_{g}} \sum_{j=1}^{N_{r}} i^{2}p(i,j|\theta)}{\sum_{i=1}^{N_{g}} \sum_{j=1}^{N_{r}} p(i,j|\theta)}$$

8. **Short Run Low Gray Level Emphasis (SRLGLE)**

$$SRLGLE=\frac{\sum_{i=1}^{N_{g}} \sum_{j=1}^{N_{r}} \left[ \frac{p(i,j|\theta)}{i^{2}j^{2}} \right]}{\sum_{i=1}^{N_{g}} \sum_{j=1}^{N_{r}} p(i,j|\theta)}$$

9. **Short Run High Gray Level Emphasis (SRHGLE)**

$$SRHGLE=\frac{\sum_{i=1}^{N_{g}} \sum_{j=1}^{N_{r}} \left[ \frac{p(i,j|\theta)i^{2}}{j^{2}} \right]}{\sum_{i=1}^{N_{g}} \sum_{j=1}^{N_{r}} p(i,j|\theta)}$$

10. **Long Run Low Gray Level Emphasis (LRLGLE)**

$$LRLGLE=\frac{\sum_{i=1}^{N_{g}} \sum_{j=1}^{N_{r}} \left[ \frac{p(i,j|\theta)j^{2}}{i^{2}} \right]}{\sum_{i=1}^{N_{g}} \sum_{j=1}^{N_{r}} p(i,j|\theta)}$$

11. **Long Run High Gray Level Emphasis (LRHGLE)**

$$LRHGLE=\frac{\sum_{i=1}^{N_{g}} \sum_{j=1}^{N_{r}} p(i,j|\theta)i^{2}j^{2}}{\sum_{i=1}^{N_{g}} \sum_{j=1}^{N_{r}} p(i,j|\theta)}$$

***Group 4. Wavelet features: first order statistics and texture of wavelet decompositions***

Wavelet transform effectively decouples textural information by decomposing the original image, in a similar manner as Fourier analysis, in low –and high-frequencies. In this study a discrete, one-level and undecimated three-dimensional wavelet transform was applied to each image, which decomposes the original image $X$ into 8 decompositions. Consider $L$ and $H$ to be a low-pass (i.e. a scaling) and, respectively, a high-pass (i.e. a wavelet) function, and the wavelet decompositions of $X$ to be labeled as $X_{LLL}$, $X_{LLH}$,$X_{LHL}$,$X_{LHH}$,$X_{HLL}$,$X_{HLH}$, $X_{HHL}$ and $X_{HHH}$. For example, $X_{LLH}$ is then interpreted as the high-pass sub band, resulting from directional filtering of $X$ with a low-pass filter along x-direction, a low pas filter along y-direction and a high-pass filter along z-direction and is constructed as:

$$X_{LLH}\left( i,j,k \right)=\sum_{p=1}^{N_{L}} \sum_{q=1}^{N_{L}} \sum_{r=1}^{N_{H}} L\left( p \right)L\left( q \right)H\left( r \right)X(i+p,j+q,k+r)$$

Where $N_{L}$ is the length of filter $L$ and $N_{H}$ is the length of filter $H$. The other decompositions are constructed in a similar manner, applying their respective ordering of low or high-pass filtering in x, y and z-direction. Wavelet decomposition of the image $X$ is schematically depicted in **Figure S1**. Since the applied wavelet decomposition is undecimated, the size of each decomposition is equal to the original image and each decomposition is shift invariant. Because of these properties, the original tumor delineation of the gross tumor volume (GTV) can be applied directly to the decompositions after wavelet transform. In this study “Coiflet 1” wavelet was applied on the original images. For each decomposition we computed the first order statistics as described in Group 1 and the textural features as described in Group 3.


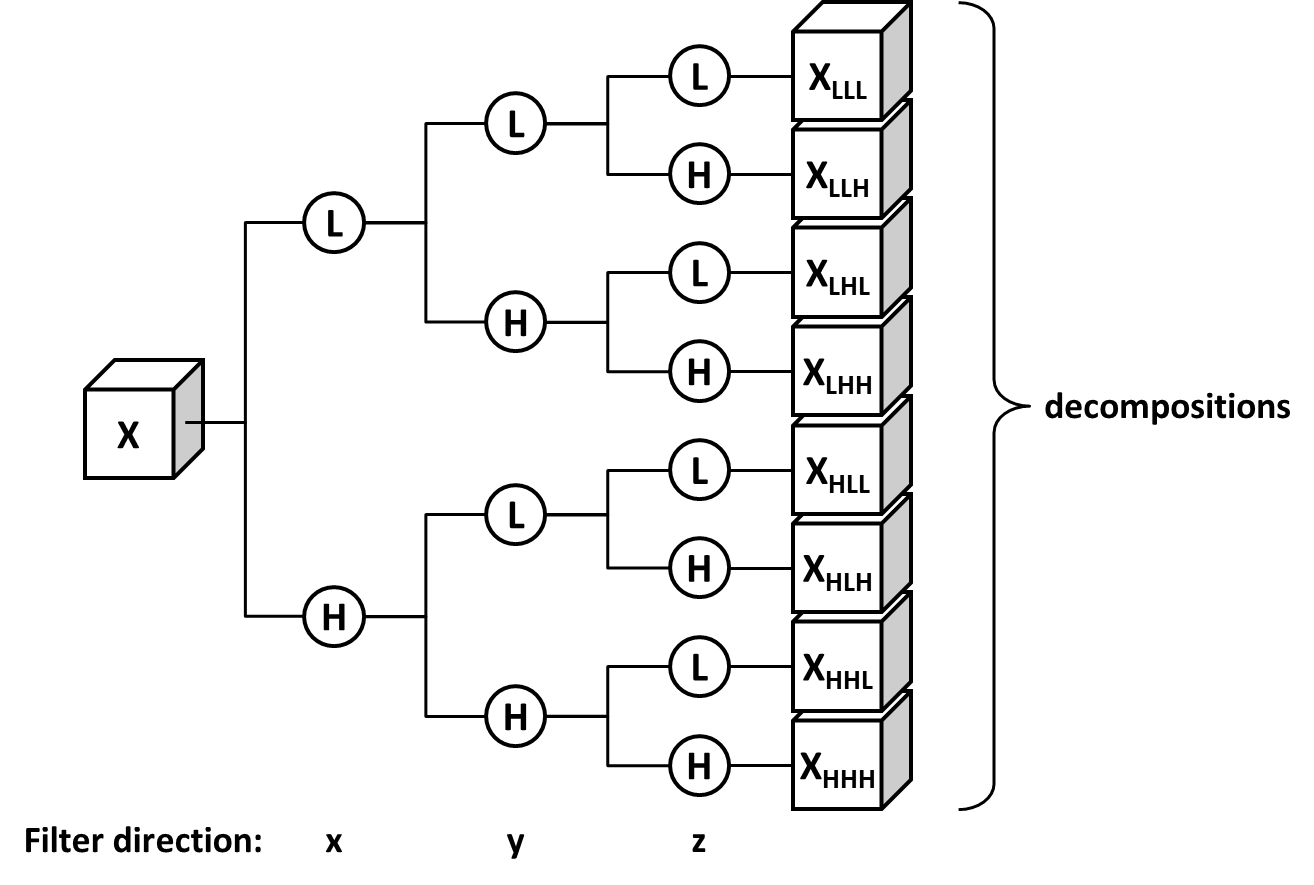


**Figure S1:** Schematic of the undecimated three dimensional wavelet transform applied to each image. The original image $X$ is decomposed into 8 decompositions, by directional low-pass (i.e. a scaling) and high-pass (i.e. a wavelet) filtering: $X_{LLL}$, $X_{LLH}$,$X_{LHL}$,$X_{LHH}$,$X_{HLL}$,$X_{HLH}$, $X_{HHL}$ and $X_{HHH}$.

**Rerences**

1. Aerts HJWL, Velazquez ER, Leijenaar RTH, Parmar C, Grossmann P, Carvalho S, Bussink J, Monshouwer R, Haibe-Kains B, Rietveld D et al: Decoding tumour phenotype by noninvasive imaging using a quantitative radiomics approach (vol 5, pg 4006, 2014). Nat Commun 2014, 5.

2. Haralick RM, Shanmugam K, Dinstein I: Textural Features of Image Classification. IEEE T Syst Man Cyb 1973, SMC-3(6):610-621.
